# Newborn physiological responses to noise in the neonatal unit^☆^^☆☆^

**DOI:** 10.1016/j.bjorl.2014.11.008

**Published:** 2015-09-07

**Authors:** Sandra Maria Schefer Cardoso, Lorena de Cássia Kozlowski, Adriana Bender Moreira de Lacerda, Jair Mendes Marques, Angela Ribas

**Affiliations:** a Communication Disorders, Universidade Tuiuti do Paraná (UTP), Curitiba, PR, Brazil; b Hospital de Clínicas do Paraná (UFPR), Curitiba, PR, Brazil; c Hearing and Language Center, Universidade Tuiuti do Paraná (UTP), Curitiba, PR, Brazil; d Department of Phonoaudiology, Universidade Tuiuti do Paraná (UTP), Curitiba, PR, Brazil

**Keywords:** Premature birth, Hearing, Neonatology, Noise effects, Noise, Recém-nascido de baixo peso, Audição, Neonatologia, Efeitos do ruído, Ruído

## Abstract

**Introduction:**

The incorporation of technologies in the care of infants has contributed to increased survival; however, this has turned neonatal unit into a noisy environment.

**Objective:**

To evaluate the physiological and functional effects resulting from the exposure to noise on low-weight newborns in incubators in a neonatal unit.

**Methods:**

Prospective, observational, quantitative, exploratory, descriptive study. The adopted statistical method included tables of frequency, descriptive statistics, and Student's *t*-test, with a 0.05 level of significance. As data collection tools, the environmental noise and the noise inside of the incubator were evaluated, and the Assessment of Preterm Infant Behavior scale was used to assess premature newborn behavior and projected specifically to document the neurobehavioral functioning of preterm infants. The data collection occurred from September of 2012 to April of 2013; 61 low-weight newborns admitted in the neonatal unit and in incubators were observed.

**Results:**

Significant differences in the variables heart rate and oxygen saturation were noted when newborns were exposed to noise.

**Conclusion:**

Low-weight neonates in incubators present physiological alterations when facing discomfort caused by environmental noise in neonatal units.

## Resumo

**Introdução:**

A incorporação de tecnologias no cuidado dos recém-nascidos contribui para o aumento da sobrevivência, porém, transformou as unidades neonatais em locais muito ruidosos.

**Objetivo:**

Avaliar os efeitos fisiológicos e funcionais decorrentes de exposição a ruídos em uma unidade neonatal em recém-nascidos de baixo peso em incubadora.

**Método:**

Estudo prospectivo, de caráter observacional, de abordagem quantitativa e natureza descritiva exploratória. O método estatístico utilizado foi o de tabelas de frequência, estatística descritiva e o teste t de student pareado, ao nível de significância de 0,05. Como instrumento de coleta dos dados foi avaliado o ruído ambiental e no interior das incubadoras e utilizada a escala assessment of preterm infant behavior para avaliação do comportamento dos bebês prematuros e projetada especificamente para documentar o funcionamento neurocomportamental dos bebês pré-termo. A coleta dos dados aconteceu no período de setembro de 2012 a abril de 2013 e foram observados 61 recém-nascidos de baixo peso em incubadora internados na unidade neonatal.

**Resultados:**

Verifica-se a existência de diferenças significativas na variável frequência cardíaca e saturação de oxigênio frente à exposição a ruídos.

**Conclusão:**

Recém-nascidos de baixo peso em incubadora apresentam alterações fisiológicas quando se encontram em desconforto causado pelo ruído ambiente de unidades neonatais.

## Introduction

Neonatal intensive care units (NICUs) are important resources for the treatment of severely or potentially severely ill patients in need of continuous and specialized care as a result of a wide variety of pathophysiological changes.^1^

The incorporation of technologies for the care of the newborn (NB) contributes to increase survival; however, this turned neonatal units (NUs) into very noisy environments.^2^

In the NU, the NB usually is in an incubator, which functions to replace the mother's womb, maintaining a warm environment and regulating temperature, depending on the NB's temperature. The incubator provides the necessary moisture for the NB's balance and protects it from infection and noise.^2^, ^3^ All care and treatment, including weighing, are carried out inside the incubator.^3^, ^4^

Excessive noise found in NUs comes from various sources, such as life support equipment, including mechanical ventilators, radiant warmers, infusion pumps, and incubators; voices/talking and movement of people in the unit; alarms; medical and family visits; handling of incubators; circulation of test equipment; careless handling when locking cabinets, drawers, trash lids, and doors; and air conditioning, among others.^5^ The side effects of the therapeutic process, in addition to the biological fragility and the long stay in those units, can increase risks to health, including hearing care.^5^, ^6^

In a NICU, environmental conditions affect the physiological and neurobehavioral status of the NB, thus the need to promote a suitable environment, acquainting the NB with it and decreasing the amount and intensity of excessive noise and light stimuli.^7^, ^8^

The noise of the equipment can affect NBs, causing increases in heart rate (HR) and respiratory rate and decreases in peripheral oxygen saturation, as well as changes in motor activity. NB placement in a noisy environment for more than 48 h is considered a risk factor for hearing loss.^9^

The Brazilian regulatory standard (norma brasileira regulamentadora [NBR]) 10152/1987 of the Brazilian Association for Technical Standards (Associação Brasileira de Normas Técnicas [ABNT]) determines 35–45 dBA (dBA–decibels with a frequency A weighting filter that approximates the corresponding auditory sensation to a isophonic curve of 40 phones) as acceptable levels for empty hospitals; the first value is the desired level, and the second the acceptable limit.^10^

Auditory rest is important to NBs for their development and growth, and to avoid responding to the stress caused by the noise, which can result in delay in weight gain and even in delay in the NB's discharge.^11^, ^12^ In addition to the damage already mentioned, if the noise reaches very high levels, it can cause hearing loss and high blood pressure (physiological effects); disturbances (psychological effects), stress, low performance, interference with oral communication, and irritability.^7^, ^11^, ^12^

The aim of this study was to evaluate the effects on physiological and functional responses resulting from noise exposure in the environment of a NU with low-birth weight infants in incubators.

## Methods

The study was approved by the Ethics Committee for Research in Humans, according to the tasks specified in Resolution CNS 196/96, under opinion No. 105.197 on September 24, 2012, and CAAE 05035912.20000.0096. Parents or guardians of all study participants spontaneously signed the informed consent.

The NB sample to participate in the study was performed by a systematic sampling technique (simplified form of random sampling). A representative sample of the number of NBs attended to in the health unit (*n* = 61) was used. Statistical analysis was performed using descriptive (mean, minimum, maximum, and standard deviation) and inferential (Student's *t*-test for paired data, at the 0.05 significance level) methods. The analyses were performed using Statistica 7.0 software.

To participate in the study, NBs were randomly chosen as they were being admitted to the NICU; the NBs were evaluated according to inclusion or exclusion criteria established for this study.

The inclusion criteria were: Informed consent signed by parents or guardians; weight between 1500 and 2500 g (a low weight by pediatric parameters); during hospitalization, continued placement in the incubator; and an otoacoustic emissions test (OAET) with a result of “approved.” The test is performed with the NB in a state of natural sleep, is painless, has no contraindications, and lasts around 10 min. For this test, a headset connected to a computer is applied to the NB's ear. The device emits low-intensity sounds and collects the results produced by the NB's ear. For this examination, the otoRead version TE (Interacoustics) equipment was used.

The exclusion criteria were: OAET with a result of “failed”; refusal of parents or guardians to participate in the study; refusal to sign informed consent; NB not within 1500–2500 g during the study period; presence of syndrome(s); and death occurring during the study.

The measurements of sound pressure levels were held at the NU in November 2012 and March 2013, according to the legal criteria established by Brazilian law, Ordinance No. 3214/78 of the Ministry of Labor and Employment, NR 17, Ergonomics, and NBR 10152/2000.^13^

The instrument used to noise evaluation was a handheld sound meter, Bruel and Kjaer Integrator brand, type 2230, with monthly calibration and owned by FUNDACENTRO/PR; the evaluation was performed by an engineer of the institution. For evaluation of sound pressure levels, the operation was conducted in compensating curve “A”, and various measurements were conducted in the morning and afternoon, by measuring the minimum and maximum levels and the mean level provided by the Leq measurement resource in NU rooms.

The measurement of environmental noise occurred in two characteristic times, as follows: (1) the usual situation in the NICU, that is, at the time of highest noise in those moments of higher flow of people, use of equipment for clinical control, shift changes, medical visits, performing procedures; (2) in quieter moments, that is, when there is less production of noise (nap time). The “nap time” in the NU is the time for complying with the NB's need for rest. There are three “nap time” moments: one in the morning, another in the afternoon, and the third at night. During “nap time”, the caretakers decrease their activities, try not to talk, the parameters of devices with alarms are decreased, and the movement of people is also diminished.

Concomitant to each measurement, noise was measured within incubators, that is, during the time of more noise in the NU, the noise was measured outside and inside the incubators, as well as at the time of least production of noise.

Data collection was conducted daily during about 30 min for each observation, and in two periods (duty shifts); thus, all times on the same day of observation were considered. As a data collection instrument, APIB (Assessment of Preterm Infant Behavior) scale validated by Als in 1982, was used. This is a widely used tool for premature NBs’ performance evaluation, designed specifically to document neurobehavioral functioning of preterm NBs.

The results were compared in the two time periods (the usual situation of the NICU and during the nap time).

As the statistical method, frequency tables and the Student's paired *t*-test were used at the 0.05 significance level (5%). The software used was Statistica version 7.0.

Sixty-one underweight NBs were observed; they weighed between 1500 and 2500 g and were in the incubator. No NBs had any kind of health problem other than low weight.

## Results

Table 1 lists the results of the descriptive statistics of the variables under analysis: Leq noise (mean level), minimum noise, maximum noise, weight, oxygen saturation (O_2_), HR, and APIB (*n* = 61), comparing two predetermined times of observation in the study methodology: quietness time (nap) and usual situation (increased production of noise). This table lists the mean, minimum, and maximum of variables and the standard deviation.

**Table 1:** Descriptive statistics of the variables analyzed and comparison between groups: S and R (*n* = 61).

Variable	Mean		Minimum		Maximum		Standard deviation		*p*
	Sleep	Noise	Sleep	Noise	Sleep	Noise	Sleep	Noise	
Leq noise (dBa)	58.62	61.34	55.80	54.50	63.40	67.90	1.70	2.88	0.0000^a^
Min. noise (dBa)	47.80	47.30	45.30	45.10	50.00	49.80	1.21	1.02	0.0351^a^
Max. noise (dBa)	75.92	78.86	71.20	74.70	80.00	83.00	2.06	1.92	0.0000^a^
Weight (kg)	1837.15	1837.14	1487.00	1487.00	2282.50	2282.50	218.63	218.64	0.5071
HR (beats/min)	137.74	142.59	110.00	122.50	158.00	160.90	10.36	8.7	0.0000^a^
O_2_ satur. (%)	95.58	94.96	93.00	91.60	98.00	97.30	1.05	1.12	0.0000^a^
APIB	3.54	3.55	2.00	2.00	6.00	6.00	1.38	1.38	0.4579

Leq, mean; min., minimum; max., maximum; HR, heart rate; O_2_ satur., oxygen saturation; APIB, Assessment of Preterm Infant Behavior.

a Significant result. Level of significance *p* = 0.05 (5%).

With the use of the paired Student's *t*-test at the 0.05 significance level (5%), significant differences were observed among the means for the following variables: Leq noise, minimum noise, maximum noise, HR, and O_2_ saturation, that is, in face of intense noise, an increase in HR and a decrease in O_2_ saturation were observed.

As for the relationship of physiological and functional variables between the two most different times of the day (nap time and the most noisy time), it was observed that the result of this data crossing has significance among noise variables and physiological data, especially HR and oxygen saturation. For the variable HR, a variation between 110 and 160 beats per minute can be noted, which is an important change, considering the reference standard for NBs, even for underweight NBs, is a mean of 120 beats per minute.

Another variable, O_2_ saturation, which represents the percentage of inspired oxygen that reaches the more distant cells in the body, also shows a very significant variation, between 91% and 98%, considering that the normal level is 100%.

As for the registry of APIB evaluation indexes, which measure the behavioral reaction of NBs in times of rest and until active stimulation by the evaluator,^2^ a variation from 2 (very good) to 6 (moderate to poor) was computed. Therefore, it can be concluded that NBs exhibit physiological and functional changes when in a state of discomfort caused by environmental noise.

## Discussion

A key aspect in the care of a premature NB is to make an attempt to reproduce, in the NICU, those conditions experienced by the NB in the intrauterine environment, while producing sufficient appropriate incentives in order to promote development.^14^

However, in the present NU, as well as in many Brazilian NUs, the intensity of the measured noise exceeds acceptable levels.^15^, ^16^, ^17^, ^18^

In a study performed in a NU to decrease the noise level in the environment, it was found that even in significantly reduced noise levels, the remaining noise was still more intense than the recommended.^19^

The measurement at both times studied here (nap time and time of greatest noise) exceeded 45 dBA, and in the “nap time”, the result of the “minimum measurement” variable is greater than when more noise generators are functioning. This is justified with noises of impact (alarms, handling of incubators, circulation of test equipment, careless handling of cabinet locks, drawers, trash lids, doors, air conditioning, etc.) produced in moments of silence, but recorded by the sound equipment.

In 2011, the Brazilian Ministry of Health stated that NICUs show quite high noise levels, with a mean of 77.4 dB (A) for background noise, a mean of 85.8 dB (A) for noise peaks, and with a significant increase during care procedures for the NB.

In a NICU study conducted in 2011, the noise measured for 48 h achieved a mean of 65–74 dB (A).^20^ In NICUs, the desired levels should not exceed Leq = 50 dB (A), Lmin = 55 dB (A), and Lmax < 70 dB (A).^21^, ^22^

Analyzing the data of the authors’ research, the sound pressure levels in the NICU studied were: Lmin. 47.80 dB (A) and 47.30 dB (A) and Lmax. 75.92 dB (A), and 78.86 dB (A); the recorded mean was 58.62 dB (A) and 61.34 dB (A) – values greater than the recommended standard.

In a noisy environment, behavioral and physiological changes in people exposed to this phenomenon can be observed.^4^, ^6^, ^7^, ^8^, ^23^

In the literature, there is evidence of deleterious effects of high levels of sound pressure occurring in NBs, for example, higher O_2_ consumption and increased HR, which result in higher energy consumption and in a delay in weight gain.^14^, ^24^ Physiological and behavioral effects in NBs exposed to noise, such as crying, agitation, and sleep disorders, among others, are also emphasized.^25^, ^26^

When observing NBs in a NU, the principal observed changes occur in HR and oximetry; these changes were described in a study where signs of stress were noted in noisy environments, notably increased HR and decreased O_2_ saturation.^14^

Another study observed physiological effects of noise in the NICU including changes in HR, increases in blood pressure, decreases of O_2_ saturation, apnea, increased intracranial pressure, and possible immune and neuroendocrine effects, in addition to behavioral and cognitive changes.^21^

The present study had the following results: increased HR (*p* = 0.0000 and standard deviation of 10.36, when sleeping and 8.7 in noise, respectively) and decrease of O_2_ saturation in NBs in incubators, in the presence of higher environmental noise levels (*p* = 0.0000 and a standard deviation of 1.05 and of 1.12, when sleeping and in noise, respectively).

Based on the abovementioned studies, it is inferred that the NBs observed in this study responded physiologically, in line with literature findings.

As for the use of APIB for observing the behavior of NBs in incubators in the NICU used in this study, this tool enabled a very straightforward method of scoring the NB's behavior and the NB's ability to adapt to new situations. As a result, a variation in the score given to adaptive behavior occurred, from 2 (very good) to 6 (moderate to poor). It was also found that NBs with greater weight and gestational age are better able to adapt behaviorally.

Searching the literature for support for this observation, it was found that it is essential to respect the behavioral state of the NB when in deep sleep; if the NB is crying, one must fully comfort the NB before performing the manipulation.^8^ Also in this sense, the literature points out that the caregiver, when watching the NB, should be cognizant of signals of poor adaptation to the environment issued by the NB, for instance, breathing, posture, muscle tone, changes in body movement, irritability, continuous crying, diffuse sleep, and hyperarousal, among others.^8^

In the book “Universal Declaration of Rights for the Premature Baby”,^27^ Article VII states: “Every premature baby has the right to rest and one should therefore comply with its period of light and deep sleep, which will henceforth be taken as essential to its proper psychic development and its biological regulation. Interrupting randomly and irresponsibly, without due cause, the sleep of a premature baby is indicative of abuse.” And in the Article VIII: “Every premature baby has the inalienable right to silence, that allows the baby to feel as close as possible to the intrauterine sound environment, in respect to its thresholds and sensitivity. Any sound source that disrespects this right shall be deemed criminal, heinous, and repugnant.”

In noise level measurements in the NU studied, the maximum level measured was 71.2–83 dBA. These measurements occurred at moments of greatest excitement within the NU. Against this, a control of the ambient noise level was recommended, which should be a practice adopted by all NUs,^2^ because of the vulnerability of the assisted clientele.

Considering the high sound pressure levels found in this study and their effect on low-weight NBs in incubators, the results show the need for interventions in order to achieve the recommended sound patterns and improve care.

Some studies have been conducted in order to gain awareness on the perception of professionals working in the NICU and parents of hospitalized NBs, with respect to the existing noise in these environments,^28^ and for implementing educational programs^29^ in these places; their authors suggested the implementation of awareness raising programs.

Therefore, caretakers should rely on knowledge, planning, teamwork, motivation, lifelong learning, and feedback. Physical changes in the unit after careful planning may be one of the more easily applied aspects. The biggest challenge resides in human activity, the main noise-producing factor within the NU.^23^

It is recommended that periodic monitoring of sound pressure levels in three shifts and different days of the week is conducted.

Importantly, there is no adverse effect, known or proposed, that would inhibit or limit the adoption of sound control measures.

In this study, it is possible to search for strategies for improving the quality of life of high-risk NBs, both with regard to their hearing conservation and to minimize the psychological and physiological effects from exposure to noise.

The scientific production in the area in question is still in its infancy, because many studies measuring NU environments, or assessing the development of low-weight NBs, have been published, but later in the NBs’ lives and outside the NU. Thus, this study may be important to encourage investigators to deploy noise reduction programs in NUs in order to improve the quality of life of the NB.

## Conclusion

This study showed high sound pressure levels in the environment of the NICU and also demonstrated changes observed in NBs, which were caused by the noisy environment.

It is concluded that NBs are affected by environmental noise as shown in their physiological or functional changes, especially at moments of higher sound production.

## Conflicts of interest

The authors declare no conflicts of interest.
